# Correction: High resolution microscopy to evaluate the efficiency of surface sterilization of Zea Mays seeds

**DOI:** 10.1371/journal.pone.0294203

**Published:** 2023-11-03

**Authors:** Yalda Davoudpour, Matthias Schmidt, Federica Calabrese, Hans Hermann Richnow, Niculina Musat

There are errors in the Funding section. The correct Funding statement is: This project was carried out within the framework of the priority program SPP 2089: “Rhizosphere spatiotemporal organization—a key to rhizosphere functions” funded by the Deutsche Forschungsgemeinschaft (DFG, German Research Foundation)—Project number 403641683 (RI-903/7-1). Hans Hermann Richnow (HHR) received the fund for this project from DFG. This work was conducted at ProVIS Centre for Chemical Microscopy (established with funds provided by Europäischer Fonds für regionale Entwicklung (EFRE) und dem Freistaat Sachsen Program) at Helmholtz Centre for Environmental Research—UFZ.

The authors apologize for this error and state that this error does not affect the results, conclusions, and overall scientific understanding of the article in any way.
