# Supplementary material for: High resolution microscopy to evaluate the efficiency of surface sterilization of Zea Mays seeds
Source: PLoS One. 2020 Nov 30;15(11):e0242247. doi: 10.1371/journal.pone.0242247 (PMC7703986; doi:10.1371/journal.pone.0242247)
Supplement: Attachment — Submitted filename: POLS_article_Rev(0820).pdf [file pone.0242247.s001.pdf]

The article, entitled “High resolution microscopy to evaluate the efficiency of surface sterilization of Zea Mays seeds,” by Yalda Davoudpour et al, for publication in POLS ONE, has some interesting results. Their approach, applying optical and scanning electron microscopic imaging as the major tools, has the potential to be developed into routine characterization protocol. There are several flaws and insufficiencies that need to be addressed properly.

Therefore, I recommend publication once those issues (details below) are addressed.

In the methods section, authors mentioned roots were cut to approximately 1 cm for optical microscopy. Also, they show images of grown roots in several images within Figs 2, 3, and 5. It will be helpful to readers if these authors give the diameter of those Petri dishes so one has a sense of actual length of those grown roots.

I am concerned with poor image quality in Figs 2, 3 and 5. It could be the result of a poor production of my copy only. Anyway, even with a cell phone one surely can obtain much higher quality images of this nature. Authors should make sure their original pictures do have adequate contrast to show clearly grown roots.

Fig. 8b needs improvement on how to present it. Its background at lower right quarter is too bright, overwhelming/dominating the lone fluorescent dot in the upper left quarter.

Images in Fig. 10 are very good.

Author’s comments about unable to determine whether microorganisms were on root surface with optical microscopy was a misconception. Optical microscopic imaging is capable do even 3-D sectional imaging, for example with confocal microscopy. Optical lenses form planar images of objects on a plane. At the resolution of their work, there is no doubt that images in Fig. 10 are features on root surface, not internal.

Fig. 11 is actually puzzling.

Those “dots,” deemed detected microorganisms, are not clear et all and are too small. The resolution of HIM should be far better. These authors should show enlarged section of those dots so that their 3-D structure can be elucidated. Images in Fig.11 do not support claims of any detected microorganism. Structures of root hairs are apparent. Red circles are very faint to almost invisible. I only saw a few random and very blurred red dots, no arrows at all.

These authors need to provide details on “cell counting,” both on methods and actual work. By imaging, readers understand how to literally count fluorescent dots in an image. This direct method gives numbers (of cells/microorganisms or else) per unit area. How to convert it to numbers per unit volume is not clear at all. These authors need to have one paragraph on methods. If literally counting “cells” on images, it is more scientific to provide the total number before a conversion to the unit number. In terms of counting, if it is manual, these authors should state so and report on how time-consuming the process actually is.
